# Plasma biomarkers of clinical response during chemotherapy plus combination antiretroviral therapy (cART) in HIV+ patients with advanced Kaposi sarcoma

**DOI:** 10.18632/oncotarget.4571

**Published:** 2015-07-09

**Authors:** Rosamaria Tedeschi, Ettore Bidoli, Maria Teresa Bortolin, Ornella Schioppa, Emanuela Vaccher, Paolo De Paoli

**Affiliations:** ^1^ Microbiology-Immunology and Virology Unit, Centro di Riferimento Oncologico, IRCCS, 33081 Aviano, Italy; ^2^ Epidemiology and Biostatistic Unit, Centro di Riferimento Oncologico, IRCCS, 33081 Aviano, Italy; ^3^ Medical Oncology A, Centro di Riferimento Oncologico, IRCCS, 33081 Aviano, Italy; ^4^ Scientific Directorate, Centro di Riferimento Oncologico, IRCCS, 33081 Aviano, Italy

**Keywords:** Kaposi sarcoma (KS), combination antiretroviral therapy (cART), G-CSF, HGF, endoglin

## Abstract

This study aimed to evaluate plasma concentration of selected cancer-associated inflammatory and immune-modulated cytokines in HIV+ patients with advanced Kaposi sarcoma (KS), and to explore candidate biomarkers capable of predicting clinical outcome in response to chemotherapy (CT) plus combination antiretroviral therapy (cART).

Thirty-seven plasma cytokines/chemokines were assessed by Luminex technology in 27 consecutive HIV+ KS patients, followed-up during CT and cART of maintanence (m-cART). Associations between plasma concentration of biomarkers and patient clinical response to m-cART were evaluated by means of Hazard Ratios (HRs) and corresponding 95% Confidence Intervals (CIs).

Plasma baseline concentration of Granulocyte colony-stimulating factor (G-CSF), Hepatocyte growth factor (HGF) and endoglin were found to be associated with m-cART clinical response (HR:1.56, 95%CI:1.09–2.22, *p* = 0.01; HR:0.32, 95% CI:0.10–0.99, *p* = 0.05; HR:0.72, 95% CI:0.54–0.96, *p* = 0.03, respectively). The multivariate analysis confirmed the associations of baseline plasma G-CSF and HGF concentration with m-cART clinical complete remission response (HR:1.78, 95% CI:1.15–2.74, *p* = 0.009; HR:0.19, 95% CI:0.04–0.95, *p* = 0.04).

Our exploratory study suggested that plasma G-CSF, HGF and endoglin may be novel predictors of clinical response during m-cART in HIV+ KS patients. Nonetheless, these findings should be further validated in an independent population study.

## INTRODUCTION

Kaposi sarcoma (KS) is the most frequent malignant lesion in patients with AIDS, even after the widespread use of combination antiretroviral therapy (cART), and it is characterized by spindle cell proliferation, inflammatory cell infiltration, angiogenesis, edema, and invasiveness [1, 2]. The complex aspect of this disease is probably supported by multiple concomitant pathogenetic factors. Today, the most favoured model is that inflammatory cytokines and chemokines, possibly up-regulated by the Tat protein of HIV or the Kaposi sarcoma related herpesvirus (KSHV) infection, induce the expression of growth factors and act synergistically in promoting and sustaining cell proliferation [3, 4]. These involved biological factors can be released and they are detectable in the blood, mirroring the tumour microenvironment. The measurement of candidates prognostic disease soluble biomarkers is rapidly evolving. Luminex multiparametric techonology has already been employed, in the setting of HIV infection and related tumours and for the prediction of patient's clinical outcome [5, 6, 7].

KS treatment choice considers the tumour extent and growth, the patient's symptoms, the immunological and virological conditions, the opportunistic co-infections, and the related complications [8, 9]. The use of cART and effective control of HIV viremia may represent the first treatment choice in case of slowly progressive disease, while chemotherapy (CT) plus cART is indicated for visceral and/or rapidly progressive disease [8]. Maintenance (m)-cART after debulking CT has been described as an effective anti-KS treatment [10, 11]. Although the survival of HIV+ patients on cART has substantially improved, long-term and cumulative CT-related immunodeficiency play a critical role in the long-term management of patients with KSHV-related advanced diseases [12], with an increased risk of death. Moreover, KSHV infection cannot be eradicated but long-term remission is possible, especially in patients whose immune system can be modulated by cART. Improved and innovative therapies are urgently needed along with new and non-invasive biomarkers to help in the identification of patients with poor clinical response who could be candidates for different or additional treatments.

In this exploratory clinical study, circulating cytokines and other signalling molecules, involved in angiogenesis, inflammation and tumour growth, were measured among HIV+ advanced KS patients before CT and during maintenance therapy. The study tested the hypothesis that candidate non invasive biomarkers were associated with KS patients clinical response to m-cART.

## RESULTS

### Patients characteristics

Blood samples for the 27 KS HIV+ patients before starting debulking CT were available; baseline plasma concentrations for the 37 immunological biomarkers and routinely evaluated HIV RNA, KSHV DNA and CD4/CD8 cell counts are shown in Table 1. At baseline, KSHV DNA was detectable in 17/27 patients with a median viral load of 50 (range: 0–8247) copies/ml, and HIV RNA in 12/27 patients with a median viral load of 170 (range: 49–257267) copies/ml. The evaluated baseline immunological status was as follows: CD4 cell counts 16.9 (range: 1.7–42.8) %, with median value of 188 (range: 7–1036) cells/mm^3^; CD8 cell counts 53.9 (range: 38.9–74.5) %, with median value of 860 (range: 279–2205) cells/mm^3^.

**Table 1:** Baseline immunological and virological characteristics of 27 consecutive HIV+ patients with advanced KS

	Median (min-max)
HIV RNA c/ml	170 (49–257267)
KSHV DNA c/ml	50 (0–8247)
CD4 %	16.9 (1.7–42.8)
CD4 cells/μL	188 (7–1036)
CD8 %	53.9 (38.9–74.5)
CD8 cells/μL	860 (279–2205)
	
IFN-γ pg/ml	9.9 (1.3–76.3)
IL-4 pg/ml	0.5 (0.5–49.7)
IL-12(p40) pg/ml	19.2 (0.5–104.7)
MIP-1β pg/ml	31.3 (12.6–187.7)
IL-1β pg/ml	0.8 (0.5–34.8)
IL-6 pg/ml	2.3 (0.5–30.9)
IL-13 pg/ml	0.5 (0.5–22.8)
TGF-α pg/ml	2.2 (0.6–8.7)
FGF-2 pg/ml	115.9 (12.2–415.3)
IL-1RA pg/ml	77.4 (11.9–659.2)
IL-7 pg/ml	11.3 (3.8–28.0)
IL-17A pg/ml	4.7 (0.8–43.4)
TNF-α pg/ml	14.4 (6.1–47.0)
IFN-α2 pg/ml	38.3 (0.5–183.7)
IL-2 pg/ml	0.5 (0.3–12.2)
IL-8 pg/ml	5.0 (1.2–75.4)
MIP-1α pg/ml	4.9 (0.5–22.8)
VEGF pg/ml	160.7 (59.1–783.8)
PLGF pg/ml	13.8 (1.3–61.2)
HB-EGF pg/ml	28.8 (1.3–124.8)
HGF pg/ml	111.3 (27.3–687.6)
Follistatin pg/ml	264.0 (85.2–986.1)
Leptin pg/ml	1377.9 (137.1–8818.2)
Endothelin-1 pg/ml	2.6 (2.6–2.6)
Endoglin pg/ml	1113.3 (584.1–1987.5)
BMP-9 pg/ml	32.7 (2.6–108.3)
G-CSF pg/ml	156.9 (13.6–646.5)
EGF pg/ml	28.8 (2.6–287.0)
Angiopoietin-2 pg/ml	1757.7 (500.7–25584.0)
MMP-3 pg/ml	5086.2 (1161.8–13724.1)
MMP-12 pg/ml	2666.2 (239.0–7584.8)
MMP-13 pg/ml	365.8 (2.3–1241.6)
MMP-1 pg/ml	3554.0 (926.0–30700.0)
MMP-2 pg/ml	57928.0 (40880.0–93422.0)
MMP-7 pg/ml	6858.0 (3582.0–17328.0)
MMP-9 pg/ml	25116 (6254.0–195130.0)
MMP-10 pg/ml	708.0 (162.0–1986.0)

Tumour clinical responses observed, considering clinical maximum response to m-cART (at T2), were: complete remission (CR) in 13/27 patients (48.2%), 3/27 patients (11.1%) were on stable partial remission (PR), 3/27 patients (11.1%) showed new PR, and 8/27 patients (30%) showed rapid disease progression.

### Plasma biomarkers and clinical patient's response to m-cART

To evaluate the association between plasma concentrations of the 37 biomarkers and patient's clinical response to m-cART, HRs and 95% CIs were calculated for continuous variations of these immunological parameters assessed at baseline, before starting CT (T0).

Plasma concentrations of 3 out of 37 immunological biomarkers were found to be significantly associated to m-cART clinical response of KS HIV+ patients (Table 2). In particular, a statistically significant association with unfavourable clinical response to m-cART was found for G-CSF plasma concentration (HR:1.56, 95% CI:1.09–2.22; *p* = 0.01). Plasma concentrations of HGF (HR:0.32, 95% CI:0.10–0.99; *p* = 0.05) and of endoglin (HR:0.72, 95% CI:0.54–0.96; *p* = 0.03) were associated to favourable clinical response to m-cART.

**Table 2:** Hazard Ratios^a^ (HR) and corresponding 95% Confidence Intervals (CI) of response to m-cART in 27 consecutive HIV+ advanced KS patients, according to the immunological plasma biomarkers, measured at baseline and found to be statistically significant

Variable	Concentration (median, pg/ml)	Range	HR^b^	95% CI	*p*-value	HR^c^	95% CI	*p*-value
G-CSF	157	14–647	1.56	1.09–2.22	0.01	1.78	1.15–2.74	0.009
HGF	111	27–688	0.32	0.10–0.99	0.05	0.19	0.04–0.95	0.04
Endoglin	1113	584–1988	0.72	0.54–0.96	0.03	0.76	0.55–1.05	0.09

a HR for a 100-fold increment of biomarker concentration.

b HRs adjusted for baseline HIV RNA and KSHV DNA levels.

c HRs adjusted for baseline HIVRNA, KSHV DNA levels and the three statistically significant biomarkers.

The associations of plasma G-CSF and HGF concentrations with patient's complete remission response during m-cART were further confirmed by the multivariate analysis (HR: 1.78, 95% CI: 1.15–2.74, *p* = 0.009; HR: 0.19, 95% CI: 0.04–0.95, *p* = 0.04) (Table 2).

Plasma concentrations of the other cytokines/chemokines, angiogenesis/growth factors or metalloproteinases assessed at T0 did not show any statistically significant association with patient's response to the treatment.

**Figure 1:**
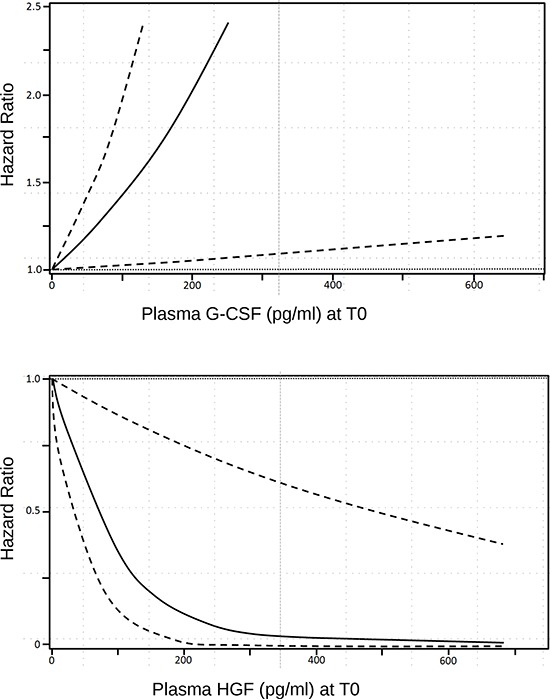
Smoothing spline plot of unadjusted hazard ratios (HRs) for clinical response to m-cART in HIV+ patients with advanced KS, according to continuous G-CSF and HGF baseline plasma concentration Hatched lines: 95% confidence intervals.

Plasma concentrations of G-CSF and HGF were plotted and represented in Figure 1. The HRs for patient response to m-cART decreased continuously, without a precise identified threshold value, with HGF plasma concentration increases (with the upper limit of the 95% CI constantly below 1.0), whereas HRs increased continuously with G-CSF plasma concentration increases.

Further, the overall median duration of response to m-cART was 45.9 months (not shown). Median responses to m-cART according to the concentration of the three plasma biomarkers measured at baseline and found to be statistically significant were: 52.1 months for G-CSF <157 pg/ml (vs. 24.3 months for ≥157 pg/ml), 54.2 months for HGF ≥111 pg/ml (vs. 11.3 months for <111 pg/ml), 54.2 months for endoglin ≥1113 pg/ml (vs. 10.2 months for <1113 pg/ml). All Log-rank tests were statistically significant (Table 3).

**Table 3:** Median response to m-cART according to the concentration of the immunological plasma biomarkers, measured at baseline and found to be statistically significant

Variable	Median response to m-cART (months)	Log-rank	*p*-value
G-CSF (pg/ml)			
<157	52.1	4.53	0.03
≥157	24.3		
HGF (pg/ml)			
<111	11.3	4.57	0.03
≥111	54.2		
Endoglin (pg/ml)			
<1113	10.2	8.59	0.003
≥1113	54.2		

Moreover, when evaluating, at T1 or T2, plasma concentrations of G-CSF, endoglin, HGF, no statistically significant changes emerged between patients who were clinically responders to m-cART and those who were not (data not shown), likewise for HIV RNA or KSHV DNA viral loads or CD4 cell counts.

## DISCUSSION

Identification of predictive and prognostic biomarkers for patients with different diseases and undergoing different therapeutic options is a very active area of investigation. Multiplex cytokines, chemokines, angiogenetic and growth factors were evaluated in a group of consecutive HIV+ patients with advanced KS, who were treated with CT plus cART followed by m-cART. Baseline plasma concentrations of G-CSF, HGF and endoglin were associated to clinical treatment response. At the multivariate analysis, G-CSF and HGF baseline plasma concentrations were confirmed to be associated to clinical complete remission response to m-cART.

For KSHV-related diseases, measurement of circulating biomarkers for the prediction of clinical outcome is still at an early stage but rapidly evolving [7]. We previously identified KSHV viral load as a prognostic factor of clinical outcome in KSHV-related lymphoproliferative disorders [13]. In contrast, in this KS group of patients on m-cART, neither KSHV DNA nor CD4/CD8 cell counts were associated with patient's clinical outcome. Similarly, in an AIDS Malignancy Consortium study on rapamycin with HAART, no significant changes in IL-6 and VEGF plasma concentrations, viral load and CD4 counts were observed during KS monitoring [14]. In a phase I trial of the MMP inhibitor COL-3, a significant decline in plasma levels of MMP-2 and MMP-9 was reported in AIDS related KS responders [15]. In a recent phase II trial of Imatinib, no correlation was found between KS-AIDS treatment response and changes in any of the different evaluated cytokines [16].

The specific roles of each biomarker are complex and multifactorial, considering that the cytokines involved in KS disease modulate multiple immune, inflammatory and other kind of responses. They are provided by chronically activated cells of the immune system or in autocrine/paracrine manner by the neoplastic cells themselves. Nonetheless, the biomarkers found to be correlated with HIV+ KS patients clinical outcome were three well defined growth factors with emerging biological roles.

Endoglin is a membrane glycoprotein, co-receptor for the transforming growth factor beta (TGF-β) superfamily, which preferentially binds TGF- β1 and TGF- β3. This protein is highly expressed by activating proliferating cells with a key function in angiogenesis [17]. In AIDS KS patients, endoglin stains were positive in the endothelium of tumour associated vessels of most KS biopsies [18]. Membrane endoglin is cleaved in the juxtamembrane region by the membrane anchored matrix metalloprotease MMP14 to release soluble endoglin [19]. Increased levels of circulating soluble endoglin were linked to poor prognosis in different human tumours [20, 21, 22, 23]. However, the function of soluble endoglin in carcinogenesis is still debated, though an antiangiogenic role in the tumour microenvironment was suggested, based on its *in vitro* capability of reducing both spontaneous and VEGF-induced angiogenesis [19]. Disturbance in the balance between membrane-localized and soluble form may occur in pathologic conditions, such as tumour characterized by angiogenic activity, like KS. The local regulation of endoglin shedding, mediated by regulation of endothelial MMP-14 expression, could result in remarkable changes in soluble endoglin concentration in the tumour microenviroment, with consequent effects on the angiogenic potential of tumour-associated endothelial cells. We could, therefore, speculate that plasma endoglin concentration of AIDS KS patients may result from an increased endoglin cleavage from membrane endoglin of the tumour cells and decreased membrane localization. The associated complete remission response during m-cART could be then related to the inhibition of angiogenesis induced by soluble endoglin, thus supporting, also in KS setting, its proposed anti-angiogenic action [19].

HGF and its Met receptor are involved in the pathogenesis of KS whose biological features are compatible with the biological properties of HGF for invasive growth, tumour proliferation, and neovascularisation [24, 25, 26]. Moreover, multiple and more complex events with different biological effects are controlled also by the cooperative interaction of the biomarkers. A recent report proposed an intriguing interaction between endoglin and HGF, showing that soluble endoglin inhibited baseline and HGF stimulated Met signalling, impairing proliferation, migration and invasion of spindle cells, in mouse tumour model [27]. According to this hypothesis, high plasma levels of endoglin and HGF detected in HIV+ KS patients associated with better outcome may behave as suppressors of KS malignancy, with consequent diminished proliferation and angiogenesis. Then again, our HIV+ KS patients might be characterised by a down regulation of Met receptor in relation to the HIV status or according to the type of KS lesion and progression stage [28]; therefore, it resulted in an impaired receptor interaction despite the high baseline HGF plasma levels.

G-CSF belongs to the family of glycoprotein molecules that stimulate the production of white blood cells, particularly granulocytes, and also mobilizes hematopoietic stem cells into peripheral blood [29]. Moreover, G-CSF acts as promoter of tumour growth through stimulation of tumour-associated angiogenesis, by increasing mobilization of endothelial progenitor cells into peripheral circulation from the bone marrow [30, 31]. This action may in part support the prognostic negative role of the high baseline plasma G-CSF concentration of HIV+ advanced KS patients. The pharmacological administration of G-CSF after chemotherapy, in the setting of HIV-related diseases, mostly with pre-existing myelosuppression, has shown clinical benefit with partial restoration of immune response [32]. However, in the current era of specific antineoplastic treatment for KS, therapy is well tolerated and usually no colony stimulating factors are required to support neutrophil counts in these patients. On the other hand, the indications that G-CSF can recruit potential KS progenitors and induce spindle cell differentiation and proliferation, indeed, open a new scenario in the cellular origin of malignant cell phenotype, pathobiology, and KS based therapies [33]. Plasma high levels of this growth factor may also be related to the cytokines alterations and to switching profiles that may influence HIV-related disease progression and also contribute to the severity of CD4 cell depletion. A tolerable ratio CD4/CD8 characterized the study patients, but a negative correlation of plasma G-CSF concentration to CD4 cell counts (data not shown) was found, though not statistically significant, similar to the inverse association reported by Shebl et al. [34].

The small sample size and length of patient follow-up represent the main weakness of our exploratory study. In addition, although several biomarkers were screened, we could not array matched healthy donors and/or HIV+ non KS patients to be evaluated in parallel. Despite these limits, our results showed that multiplexed analysis of plasma biomarkers is useful for the evaluation of prognostic markers of clinical outcome and of potential predictive markers of treatment response that may support the clinical management of HIV+ KS advanced patients. Being this a small pilot study designed to assess feasibility, our findings are suggestive rather than definitive. Nonetheless, the observed association of three plasma biomarkers justifies the validation of these findings in a large population study.

## MATERIALS AND METHODS

### Study population

Twenty seven consecutive HIV+ patients with advanced KS stage (≥T1), according to the AIDS Clinical Trials Group (ACTG) classification [35], were considered in this study. All patients were of Caucasian ethnicity, all males, except for one female, with a median age of 41 (range: 35–52) years. They were followed-up at the Department of Oncology & AIDS at our Institute, during the period 1997–2012. Since then, they all received Protease Inhibitors (PI)-based cART. They were part of an ongoing phase II study and received 6 cycles of Liposomal Doxorubicin plus CT, followed by cART as maintenance anti-KS treatment (m-cART).

Clinical evaluation of treatment response was performed after the end of debulking CT and every two months during m-cART. Study endpoint for the evaluation of clinical and biological correlations was set at the maximum response to m-cART.

Clinical response to the therapy was recorded according to ACTG criteria [35] in the following categories: patients with complete remission (CR), partial remission (PR), stable disease (SD), and progressive disease. De novo CR developing during m-cART was arbitrarily defined as new CR.

In particular, for the statistical analysis, patients were categorized into two main groups: CR patients versus no CR patients (e.g., patients with partial remission/stable disease or progressive disease).

After each patient provided written informed consent, peripheral blood samples were collected at consecutive visits: before starting (baseline, T0) and after debulking CT (T1), and at the time of maximum clinical response during m-cART (T2).

### Laboratory methods

Whole blood was processed immediately for cytofluorimetric analysis and T lymphocyte CD4 and CD8 subsets were evaluated by a single platform whole blood lysing technique (EPICS XL flow cytofluorimeter). Plasma HIV RNA viral load was assessed by RealTime HIV-1 assay (Abbott Molecular Diagnostics, Rome, Italy). Plasma KSHV DNA viral load was measured by a TaqMan real-time PCR home made method. Our molecular assay reported a good sensitivity, with consistent and comparable KSHV DNA viral loads detected in both plasma and PBMCs specimens from patients with different KSHV-related diseases [36, 37]. Therefore, we chose the plasma method, which is also more feasible as compared to the PBMCs one, as it requires a simpler preparation of the sample and the beta globin standard curve does not need to be run. Stored plasma samples were assessed by using four different multiplex panels of cytokines/chemokines, angiogenetic, and growth factors and matrix metalloproteinases (Human Cytokine/Chemokine magnetic bead panel kit, Human Angiogenesis/Growth Factor magnetic bead panel kit, Human MMP Panel 1 and 2 magnetic bead kit, EMD Millipore Corporation, Billerica, MA). Multiplex and different experiments were performed using the Luminex-200 (Luminex Co., US), following manufacturer's instructions and as already described [5].

### Statistical analysis

Survival probabilities were estimated by means of the Kaplan-Meier method, and compared using the log-rank test. Median response to m-cart was also calculated. The association between immunological and virological biomarkers and disease progression was estimated by means of the Cox proportional hazards model. Hazard Ratios (HRs) and corresponding 95% confidence intervals (CIs) were calculated for continuous variations of the considered biomarker concentration. Analyses were firstly adjusted for HIV RNA and KSHV DNA levels. Biomarkers with a statistically significant association with clinical response to the treatment were thereafter included in a multivariate model and were then plotted. Analyses were performed by means of SAS, version 9.2 (SAS Institute Inc., Cary, NC, 2002–2008). All statistical tests were two-sided and *p*-value < 0.05 was considered statistically significant. Due to the exploratory nature of this study, no attempt was made to correct for multiplicity of analyses and nominal *p values* were reported.
